# Acclimation and size influence predation, growth, and survival of sexually produced *Diploria labyrinthiformis* used in restoration

**DOI:** 10.1038/s41598-024-73727-8

**Published:** 2024-11-01

**Authors:** Mark C. Ladd, Andrew A. Shantz, Cailin Harrell, Nicole K. Hayes, David S. Gilliam, Erinn M. Muller, Keri L. O’Neil, Brian Reckenbeil, Zachary Craig, Diego Lirman

**Affiliations:** 1 Population and Ecosystems Monitoring Division, NOAA Southeast Fisheries Science Center, Miami, FL USA; 2 Cooperative Institute for Marine and Atmospheric Research, University of Hawai’i at Mānoa, Honolulu, HI USA; 3 Florida State University, Tallahassee, FL USA; 4 Department of Marine Biology and Ecology, Rosentiel School of Marine, Atmospheric, and Earth Science, University of Miami, Miami, FL USA; 5 Nova Southeastern University Halmos College of Arts and Sciences, Hollywood, FL USA; 6 Mote Marine Laboratory, Summerland Key, FL USA; 7 Center for Conservation, The Florida Aquarium, Apollo Beach, FL USA; 8 Division of Aquatic Resources, Kailua-Kona, HI USA

**Keywords:** Stony coral tissue loss disease, Corallivory, *Diploria labyrinthiformis*, Coral restoration, Coral reef, Florida’s coral reef, Coral propagation, Restoration ecology, Conservation biology, Restoration ecology

## Abstract

Stony coral tissue loss disease (SCTLD) has swept through Florida reefs and caused mass mortality of numerous coral species. In the wake of these losses, efforts are underway to propagate coral species impacted by SCTLD and promote population recovery. However, numerous knowledge gaps must be addressed to effectively grow, outplant, and restore populations of the slower growing, massive species that were lost. Here, we used sexual recruits of *Diploria labyrinthiformis* spawned in captivity to understand how conditioning, coral size, and nutritional status at outplanting affect coral survivorship, growth, and susceptibility to predation. We found that ex situ conditioning with supplemental feeding increased coral growth rates, resulting in larger sized corals at the time of outplanting. In turn, these corals had higher growth rates in the field and a lower probability of being removed by predators than outplants that were conditioned in in situ nurseries. Additionally, we found that coral size was an important predictor of survivorship, suggesting that hastening the speed at which young corals grow and outplanting larger juveniles can improve restoration outcomes. Taken together, our results suggest that providing supplemental food to corals at ex situ facilities confers benefits that could help restore populations of massive coral species impacted by SCTLD.

**Supplementary Information:**

The online version contains supplementary material available at 10.1038/s41598-024-73727-8.

## Introduction

Coral populations on reefs across Florida and the Caribbean are experiencing a multi-year disease-related mortality event associated with stony coral tissue loss disease (SCTLD^1,2^). First detected in 2014 on reefs in Miami, Florida USA, SCTLD affects at least 21 species of coral, primarily slower growing massive and submassive species including key reef-building corals and species listed under the U.S. Endangered Species Act^3,4^. Corals affected by SCTLD can display tissue loss lesions that rapidly progress and often result in whole colony mortality^5,6^. Since 2014, SCTLD has caused mass mortality of numerous coral species, including highly susceptible species like *Diploria labyrinthiformis*, *Colpophyllia natans*, and *Orbicella*s pp., driving precipitous declines in coral populations and the ecological functions they provide^2,7^.

Coral reefs in Florida were the first to be impacted by SCTLD, with the most intense effects realized from 2014 to 2020 as SCTLD spread south from southeast Florida through the Florida Keys, decreasing the abundance of corals on already coral depauperate reefs^1,8^. In the wake of the disease front, the prevalence of SCTLD on many Florida reefs has declined to negligible levels, particularly in northern regions where SCTLD was first observed^8,9^, prompting efforts to actively restore populations of coral species impacted by SCTLD. Additionally, decades of previous decline within these species due to prior disease outbreaks and widespread bleaching events^10–12 ^further justified the need to focus on these major reef building species for restoration of reef function^13^. To meet this need, coral restoration practitioners are scaling up the propagation of SCLTD-susceptible coral species in ex situ and in situ nurseries to produce sufficient numbers to support restoration efforts. Recent advances in ex situ spawning of SCTLD susceptible species held in long-term living gene banks^14^ have led to an increased supply of sexually derived corals for local restoration projects. However, given limited experience propagating and outplanting these slower-growing massive coral species, numerous knowledge gaps must be addressed to effectively grow, outplant, and restore populations of these species in the wake of the SCTLD outbreak.

Corals outplanted to contemporary reefs as part of restoration efforts are likely to encounter numerous stressors ranging from acute events like thermal stress^15,16 ^and predation^17,18 ^to chronic stressors like sedimentation, competition with macroalgae^19^, and nutrient pollution^20,21^. These stressors can independently and interactively cause partial or complete coral mortality^21,22^, reducing outplant success and working against coral restoration efforts. Consequently, methods to maximize outplant survival and growth are imperative to successful coral restoration strategies. Conditioning corals under particular regimes before outplanting them to reefs may be one effective strategy to better prepare corals to cope with stressors and increase their probability of survival^23–25^. For example, *Acropora cervicornis*fragments pre-exposed to a variable temperature regime prior to two weeks of thermal stress exposure were slower to bleach and lose tissue compared to fragments held under ambient temperatures^23^. Similarly, lipid and protein reserves can help corals resist and recover from bleaching^24–26^, including increasing these energy reserves via supplemental feeding of corals before they are exposed to thermal stress^27^. Thus, conditioning that increases these energy reserves may confer some degree of resistance to outplanted corals against stressors they are likely to encounter. However, despite the recent focus on the propagation of SCTLD-susceptible species, there is a lack of information on the costs and benefits of conditioning these corals before outplanting^26^.

Intense predation by fish on massive coral species outplanted to Florida reefs has emerged as a substantial bottleneck to restoring non-*Acropora *species^17,18^. Indeed, recent work on reefs in Miami, Florida found that predators can remove > 70% of outplanted colonies within the first month, highlighting the urgent need to find effective methods to reduce predation on outplanted corals^28^. However, predation can vary widely both among species and between genotypes within a species^17,28,29^, and also vary geographically^18^. This variation in the prevalence and intensity of predation may be driven in part by differences in resource quality. Coral species and genotypes can differ in key nutritional aspects like lipid and protein content^28,30^, which may influence predator feeding decisions^31^. Additionally, basic factors like area of live tissue at outplanting can influence outplant success for coral species with branching morphologies^32^, though the importance of coral size at outplanting for corals with a mounding growth form remains largely untested (but see^33^). For instance, in Miami, Florida, clusters of *Orbicella faveolata *fragments outplanted together (~ 25 cm^2 ^live coral tissue) were less susceptible to predation than single fragments^28^ (5 cm^2^ live coral tissue), suggesting larger corals may be less susceptible to mortality from predation compared to smaller corals. Thus, while acclimation under certain conditions (e.g. supplemental feeding) may increase outplanted corals resistance to particular stressors, the same conditioning may also make a coral more vulnerable to other impacts, such as predation.

**Fig. 1:**
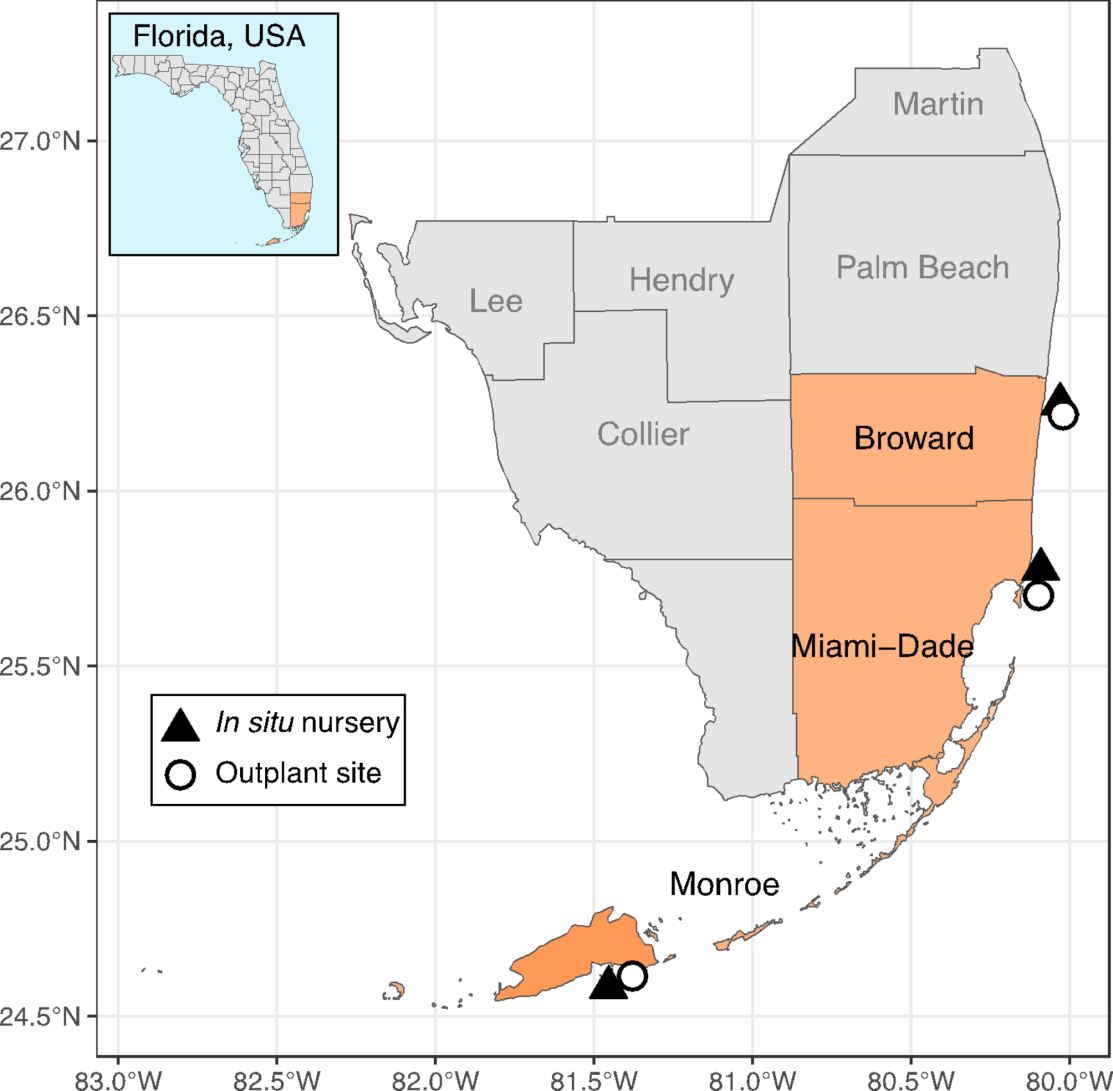
Map displaying the locations of in situ nurseries and outplant site locations in Broward, Miami-Dade, and Monroe Counties in South Florida, USA.

Here, we aimed to understand the role of conditioning, coral size (or age post-settlement), and nutritional status on coral survivorship, growth, and susceptibility to fish predation after outplanting. To do so, we reared two cohorts of *D. labyrinthiformis* spawned at the Florida Aquarium under three different conditioning treatments: (1) In Situ at different Florida nursery locations, or ex situ and (2) Fed or (3) Unfed for a 3 month pre-outplant conditioning phase. We then conducted a reciprocal outplant study at three locations spanning southeast Florida (Fig. 1) using these pre-conditioned corals to test the generalizability of any benefits conferred during the conditioning phase and better understand spatial variability in predation on outplanted corals. We tracked coral growth, survival, and changes in tissue condition prior to outplanting, as well as through 3-months after outplanting. Specifically, we focused on three major research questions pertaining to pre-outplant conditioning and post-outplant survival:

*Pre-outplant coral conditioning phase*:

1. How do different conditioning strategies and nursery types influence juvenile coral lipid and protein content, growth, and survivorship?

*Post-conditioning coral outplant phase*:

1. How does pre-outplant conditioning and coral size/age affect predation, growth, and survivorship of outplanted juvenile coral colonies?
2. How do geographic regions across south Florida vary in corallivory, survivorship, and growth of outplanted juvenile coral colonies?

## Results

### Pre-outplant coral conditioning phase

*Diploria labyrinthiformis* colonies from the 2020 cohort (18 months post-settlement; *n* = 450) had 100% survivorship during the 3-month conditioning period, regardless of conditioning treatment. Survivorship of *D. labyrinthiformis* colonies from the 2021 cohort (6 months post-settlement; *n* = 450) was ≥ 90% for all treatments. For the 2021 cohort, survivorship was 90%, 92%, and 95% for the Unfed treatment, the In Situ treatment, and the Fed treatment, respectively.

**Fig. 2:**
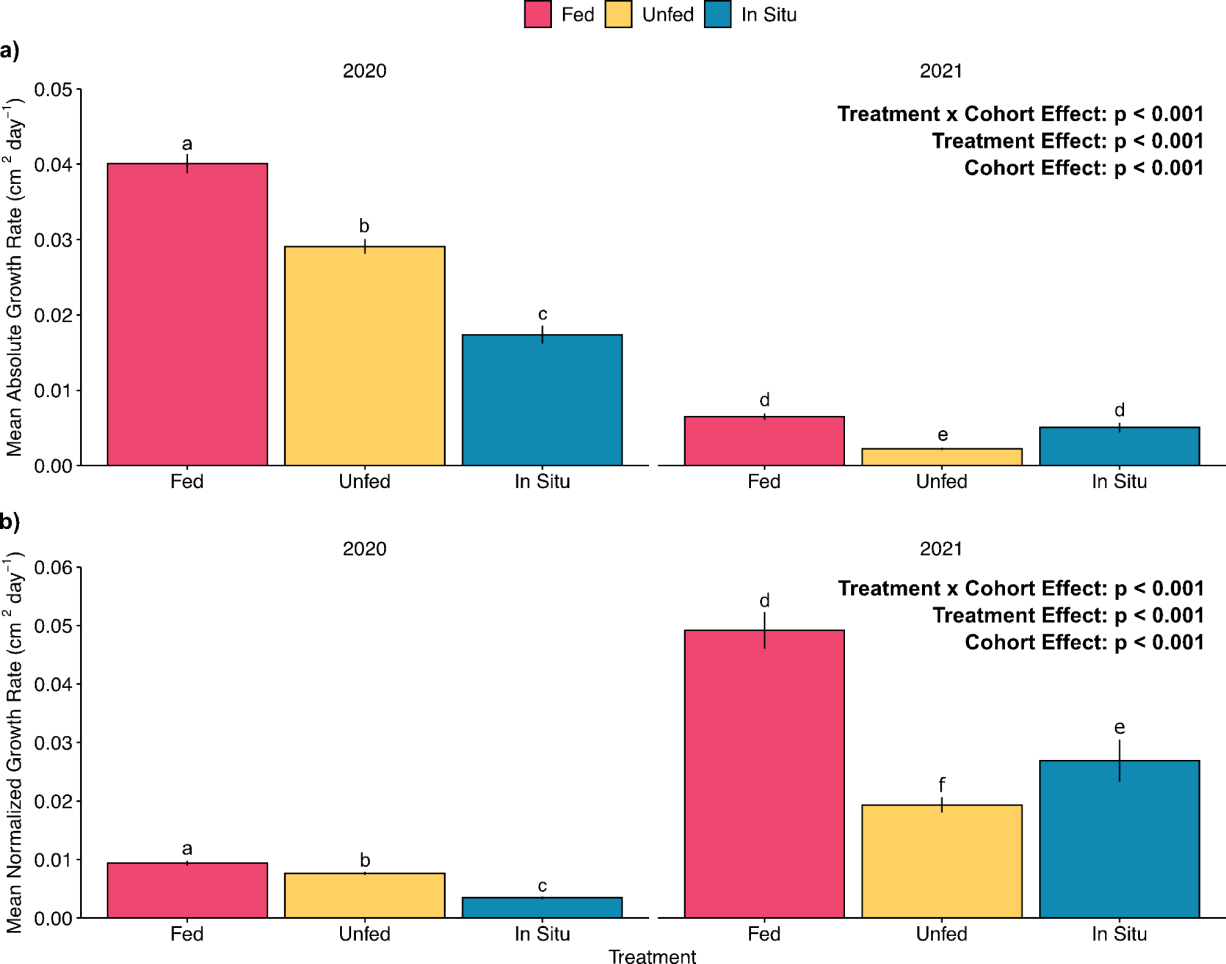
(**a**) Mean absolute growth rate (cm^2^ day^−1^) and (**b**) normalized growth rate (cm^2^ day^−1^ normalized to initial cm^2^ live tissue area) of *D. labyrinthiformis* prior to outplanting. Colonies were acclimated for 3 months under different conditions at ex situ facilities (Fed and Not Fed) and in situ nurseries (In Situ). Statistics from linear mixed effects models. Bars that share the same letter indicate a lack of statistically significant differences (*p* ≥ 0.05) between values per *post hoc* test with Tukey’s correction. In situ data from NSU nursery not included in analyses or figures due to intense predation at this location during the acclimation phase. Error bars indicate ± SE.

Absolute coral growth rates (cm^2^ day^−1^) during the conditioning phase varied widely among cohorts and treatments (LME: Cohort × Treatment effect: χ^2^(2) = 137.0, *p* < 0.001; Fig. 2a). Mean growth rates across all Cohort × Treatment combinations ranged 20-fold, from a high of 0.04 ± 0.001 (mean ± SE) cm^2^ day^−1^ in the Fed 2020 cohort to a low of 0.002 ± 0.0001 cm^2^ day^−1^ for Unfed colonies from the 2021 cohort. Within the 2020 cohort, Fed and Unfed corals reared ex situ grew 2.3× and 1.7× faster than In Situ corals, respectively. In contrast, in the 2021 cohort, Fed and In Situ corals grew faster than Unfed corals. Corals from the 2020 cohort began the experiment on average approximately 30× larger in area than 2021 cohort corals (4.6 ± 0.07 cm^2^ vs. 0.15 cm^2^ ± 0.005). Normalized growth rates (cm^2^ day^−1^ per initial cm^2^ of live tissue) also varied among cohorts and treatments during the conditioning phase (LME: Cohort × Treatment effect: χ^2^(2) = 55.40, *p* < 0.001; Fig. 2b). Here, the 2021 Fed corals increased in size at a mean rate of 0.05 ± 0.0004 cm^2^ day^−1^ per initial cm^2^ of live tissue, more than 2× faster than 2021 Unfed and In Situ corals. Corals from the 2020 cohort conditioned in situ had the lowest normalized growth rate of any Cohort × Treatment combination, increasing in size at a mean rate of 0.003 ± 0.001 cm^2^ day^−1^ per initial cm^2^ of live tissue.

### Protein and lipid content

**Fig. 3:**
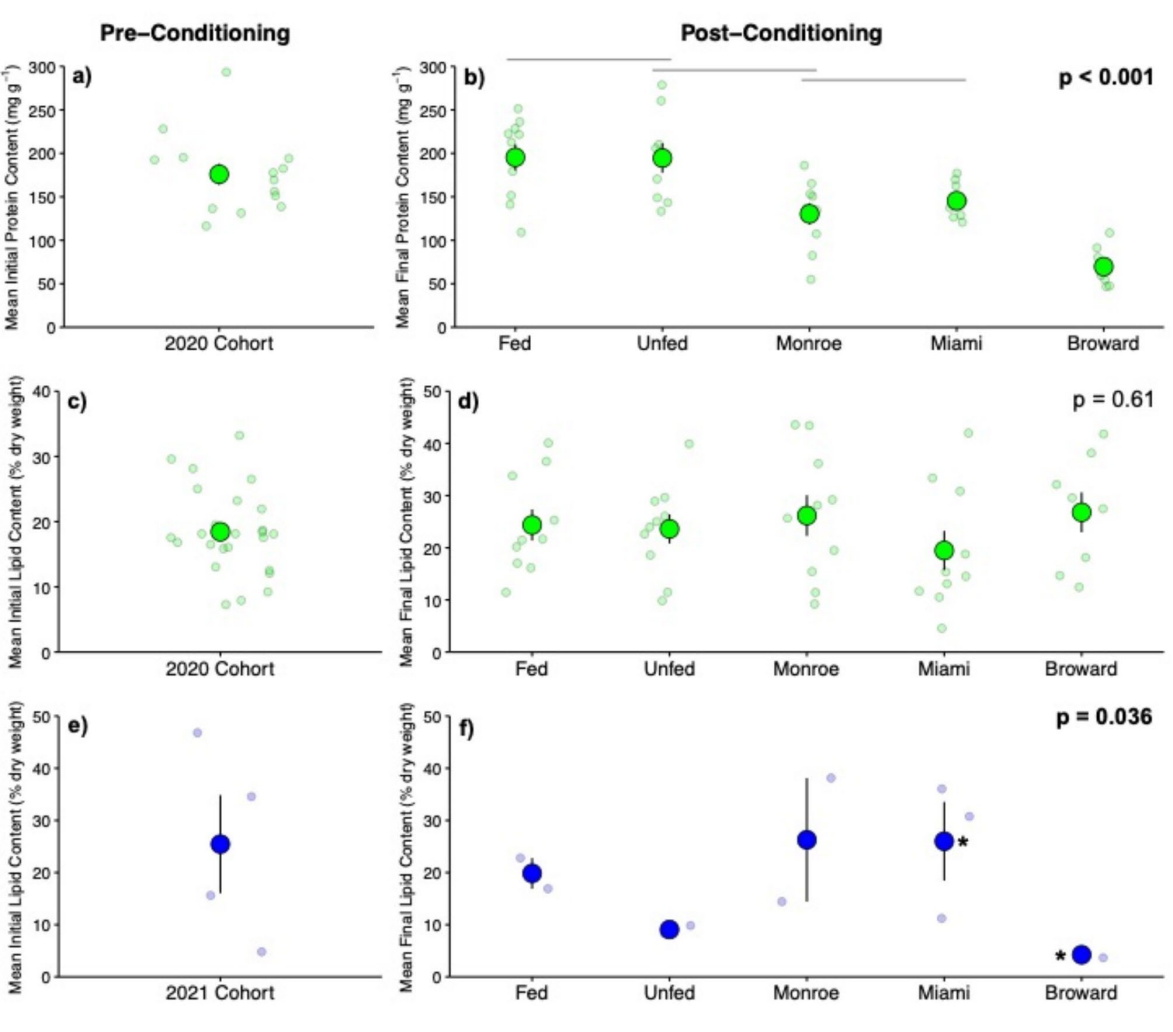
Mean protein (top row; 2020 Cohort) and lipid (middle row; 2020 Cohort and bottom row; 2021 Cohort) content from *D. labyrinthiformis* colonies before and after being reared under different conditions for 3 months. Panels (**a**), (**c**), and (**e**) display pre-conditioning values. Panels (**b**), (**d**), and (**f**) display post-conditioning values for the two ex situ treatments (Fed and Unfed) and in situ nurseries. For panel (**b**) groups with overlapping black bars are not significantly different from each other. In panel (**f**), groups with an asterisk (i.e. Miami and Broward) were significantly different from one another. Small translucent points represent raw data, larger opaque points represent mean. Error bars indicate ± SE. Statistics from one-way ANOVA, intra-group differences detected using Tukey’s HSD post hoc analysis.

After 3 months of conditioning, the total protein content of 2020 cohort corals differed significantly among in situ nursery locations and treatments (ANOVA; *p* < 0.001). Colonies reared ex situ (both Fed and Unfed) had the highest protein content, colonies reared in situ at the Broward nursery had the lowest total protein content, and colonies acclimated in situ at the Miami and Monroe nurseries contained intermediate protein levels (Fig. 3a,b). Samples from the 2021 cohort did not yield sufficient tissue to assess protein content due to small coral sizes. Lipid content in the 2020 cohort averaged 23.97 ± 1.5% of tissue weight, was highly variable, and did not differ among treatments (ANOVA; *p* > 0.61; Fig. 3c,d). In contrast, although replication was low lipid content in the 2021 cohort differed among treatments and in situ nursery locations (ANOVA; *p* = 0.04; Fig. 3e,f). Within this cohort, colonies from the Miami In Situ nursery had significantly higher lipid reserves than those grown at the Broward In Situ nursery (26% ± 7.5% vs. 4.3 ± 0.5%; Cohen’s D = 2.35). Corals conditioned under the Fed treatment had >  2× larger lipid reserves (19.85 ± 2.9%) compared to Unfed corals (9.08 ± 0.8%), though this difference was not statistically significant (ANOVA; *p* > 0.05).

## Post-conditioning outplant phase

**Fig. 4:**
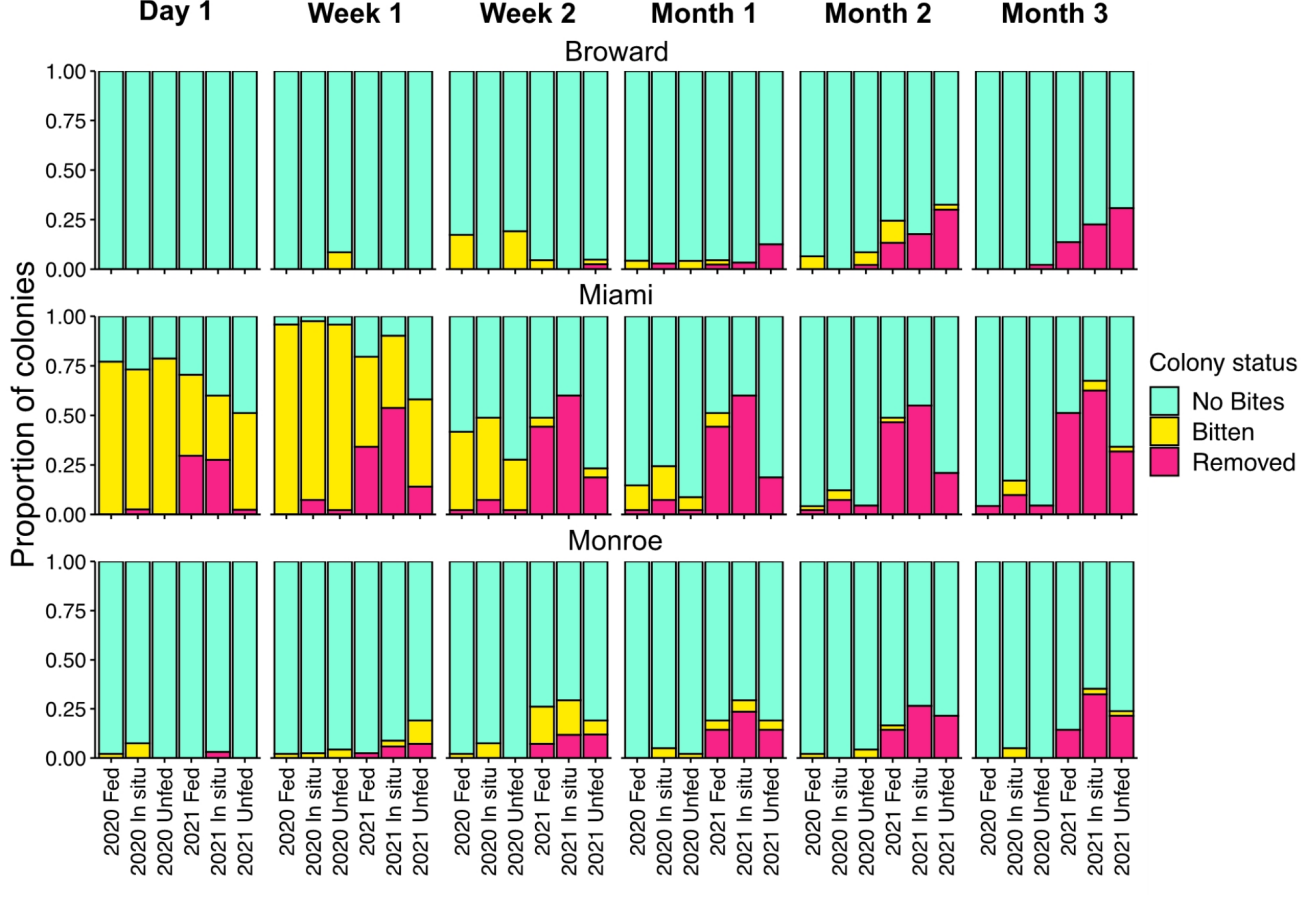
Proportion of outplanted *D. labyrinthiformis* colonies recorded as either having no bites (teal), at least 1 new bite from a predator (yellow), or completely removed (pink) at each survey for each outplant location.

During the 3 months that we tracked corals after outplanting predation intensity varied widely among outplant regions and surveys (Fig. 4). Predation was lowest at the Broward site, with < 25% of colonies in any Cohort × Treatment combination being bitten or removed by predators during the first month. The highest predation intensity was recorded at the Miami site, where 50–75% of corals were bitten or removed within the first 24 h of deployment across all Cohort × Treatment combinations. After one week of deployment at the Miami site, > 75% of all corals were either bitten or removed, except for the 2021 Unfed treatment, which had the lowest predation at this site (but still above 50%). At the Monroe site in the Lower Florida Keys, the number of corals with bites present was highest in Week 2. Across all three sites, the proportion of colonies with new bites decreased at the Month 1 survey and remained low at Month 2 and Month 3. Across all sites we observed a higher proportion of 2021 cohort corals that were completely removed by predators compared to 2020 cohort corals.

**Fig. 5:**
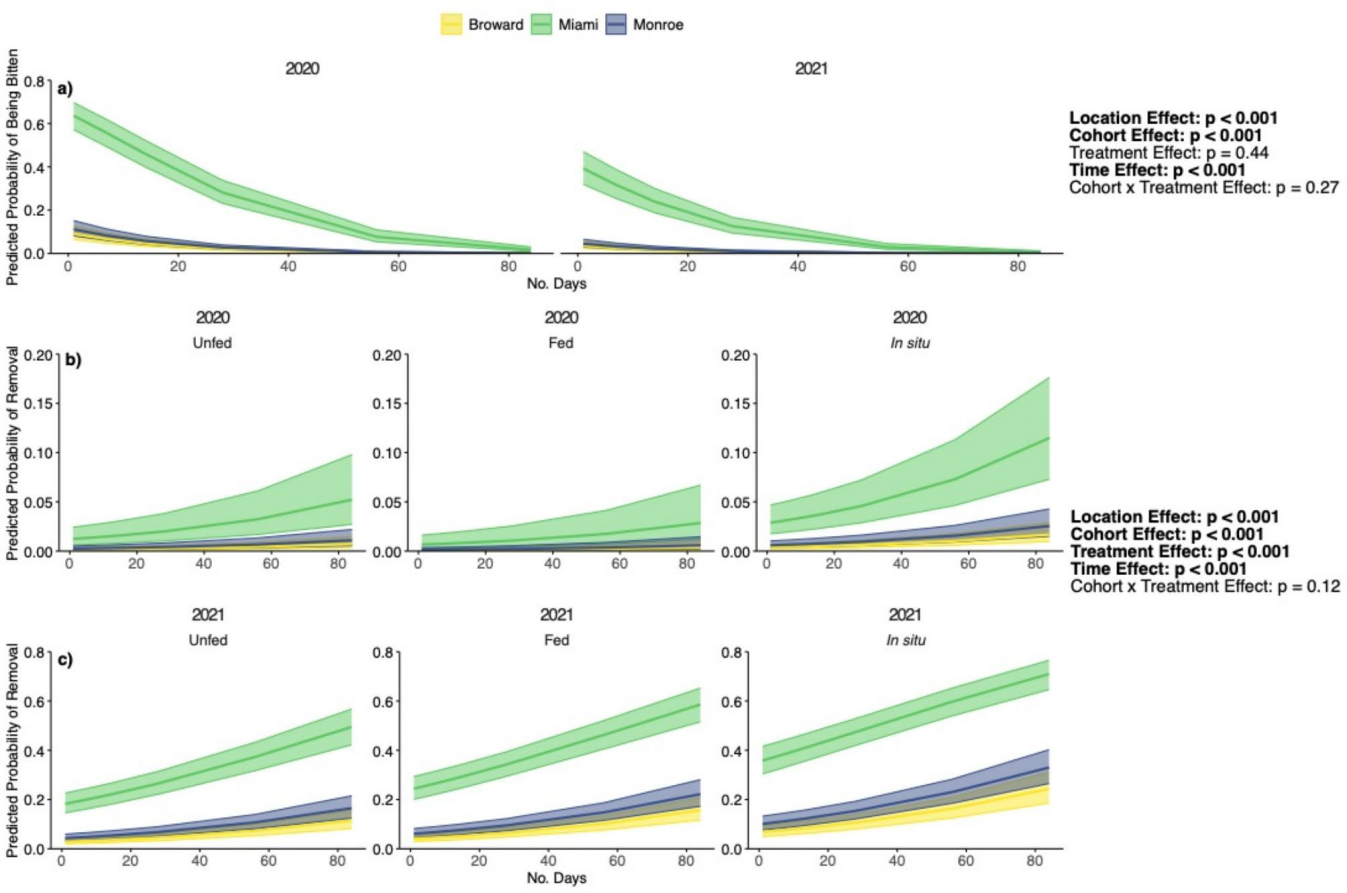
(**a**) Predicted probability of outplanted corals being bitten and (**b**,**c**) removed based on estimated marginal means derived from field surveys. Statistics from generalized linear model with binomial distribution. Shaded regions represent 95% confidence intervals. Note that y-axis scales vary between panels (**b**) and (**c**).

The probability of a coral being bitten was significantly influenced by Location (GLM: χ^2^_2_ = 616.7, *p* < 0.001), with the highest predicted probability of being bitten in Miami compared to similar predicted probabilities for corals in Monroe and Broward (Fig. 5a). The probability of being bitten was also significantly influenced by Cohort (GLM: χ^2^_1_ = 90.88, *p* < 0.001), with 2020 corals more likely to be bitten than 2021 corals and decreased through time (GLM: χ^2^_1_ = 430.3, *p* < 0.001) across Locations and Cohorts. The probability of a coral being bitten was not influenced by Treatment (χ^2^_2_ = 1.67, *p* = 0.44).

The probability of a coral being removed by predation varied across Locations (GLM: χ^2^_2_ = 266.6, *p* < 0.001), with significant differences among all regions in the predicted probability of removal (Miami > Monroe > Broward; Fig. 5b). There was a significant effect of Cohort on the probability of removal (GLM: χ^2^_2_ = 525.0, *p* < 0.001), but opposite of patterns for the probability of being bitten; corals from the 2021 cohort were more likely to be removed than 2020 cohort corals. Time was also a significant predictor of the probability of a coral being removed (GLM: χ^2^_2_ = 94.11, *p* < 0.001), with the probability of removal increasing throughout the study. Lastly, we detected a significant effect of Treatment (GLM: χ^2^_2_ = 46.20, *p* < 0.001) on the probability of a coral being removed, with post hoc tests indicating that corals acclimated in situ had a higher probability of removal than corals acclimated ex situ, regardless of if they were Fed or Unfed.

**Fig. 6:**
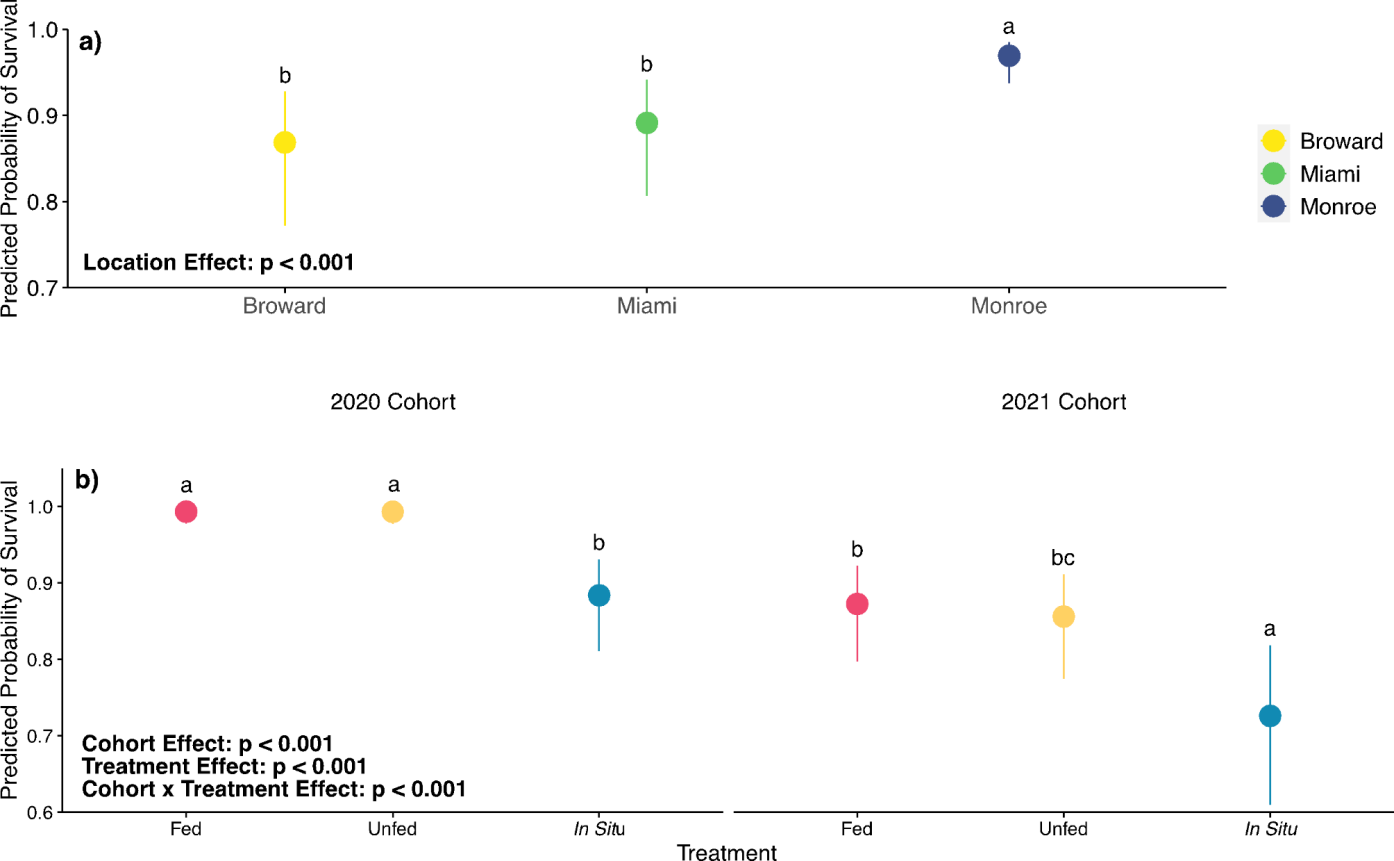
(**a**) Predicted probability of survival of *D. labyrinthiformis* colonies of 3 months of being outplanted based on estimated marginal means derived from field surveys for each of the sites included in this study. (**b**) Predicted probability of survival among each Cohort × Treatment combination at the end of the 3-month outplant period. Statistics from generalized linear model with binomial distribution. Error bars represent 95% confidence intervals. Points share the same letter indicate a lack of statistically significant differences between values within a location per *post hoc* test with Tukey’s correction.

Survivorship of outplanted *D. labyrinthiformis* colonies after 3 months in the field varied among Location, Treatment, and Cohort. We detected a significant Location effect on the predicted probability of survivorship (GLM: χ^2^_2_ = 42.5, *p* < 0.001; Fig. 6a), with post hoc tests indicating that predicted probability of survivorship across all Cohorts and Treatments was higher in Monroe compared to Miami and Broward. We also detected a significant Cohort × Treatment effect (GLM: χ^2^_2_ = 18.4, *p* < 0.001; Fig. 6b) on the probability of survivorship of outplanted colonies. Across all three regions, 2020 cohort corals consistently had higher survivorship than the 2021 cohort corals. Within each Cohort, Fed and Unfed corals had a higher probability of survivorship compared to colonies conditioned under the In Situ treatment. The 2020 Broward In Situ colonies had the highest mortality out of any Cohort × Treatment × Region combination (80%; Fig. S1). Apart from the 2020 In Situ cohort from Broward, all other 2020 Cohort × Treatment combinations had ≥ 90% survivorship. Survivorship for 2021 corals varied more widely, ranging from a high of 86% (Fed corals in Monroe) to a low of 35% (In Situ corals in Miami).

**Fig. 7:**
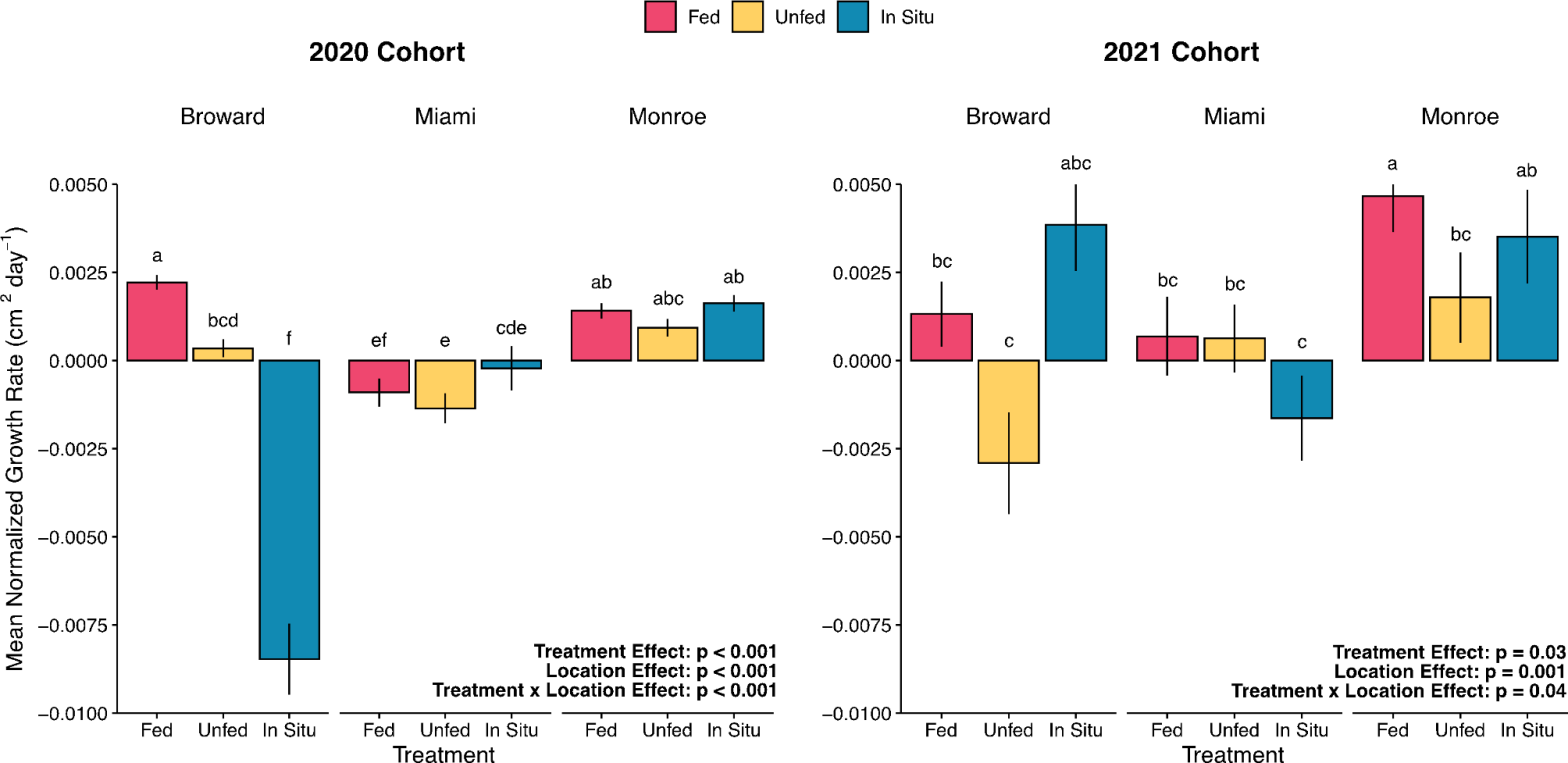
Mean normalized growth rate (cm^2^ day^−1^ normalized to initial cm^2^ live tissue area) of *D. labyrinthiformis* colonies after 3 months of being outplanted to reef sites in South Florida. Statistics from generalized linear mixed effects model. Bars that do not share the same letter indicate statistically significant differences between values within a location per post hoc test with Tukey’s correction. Error bars indicate ± SE.

Normalized growth rates (cm^2^ day^−1^ per initial cm^2^ of live tissue) of outplanted *D. labyrinthiformis* colonies from the 2020 cohort varied among Location and Treatments (LME: χ^2^_4_ = 147.0, *p* < 0.001) after 3 months of deployment in the field (Fig. 7). On average, all 2020 cohort corals outplanted in Monroe displayed positive growth, whereas corals outplanted to Miami displayed negative (i.e. loss of live tissue from predation) or no growth. In Broward, Fed corals increased in live area faster than Unfed corals, while In Situ corals displayed high levels of tissue loss, likely an artifact of intense predation while conditioning in the Broward nursery. We also detected a significant Location × Treatment effect on growth rates of 2021 cohort corals (LME: χ^2^_4_ = 10.31, *p* = 0.04), which displayed similar patterns to the 2020 cohort in Monroe and Miami. However, in Broward we found that both Fed and In Situ conditioned colonies displayed positive growth and grew faster than Unfed colonies, which displayed net loss of live tissue during the course of the experiment. Absolute growth rates (cm^2^ day^−1^) mirrored patterns observed in normalized growth rates and are provided as Fig. S2.

## Discussion

Here, we show that juvenile colonies of *Diploria labyrinthiformis* produced from ex situ sexual reproduction and reared for coral restoration benefited from ex situ conditioning before being outplanted to reefs in Florida, USA. Pre-outplant benefits of ex situ conditioning for three additional months post-settlement included increased growth rates resulting in larger sized corals at the time of outplanting, as well as increased protein content. Importantly, these pre-outplant benefits translated into improved performance when corals were outplanted to reef sites across South Florida. Specifically, after 3 months of deployment on reefs, corals conditioned ex situ had higher growth rates and a decreased probability of removal by predators compared to corals conditioned in situ. Additionally, we found that coral size was an important predictor of survivorship, suggesting that hastening the speed at which young corals can achieve larger sizes and outplanting larger sexually derived coral juveniles can benefit coral restoration efforts.

Rearing corals in ex situ systems affords practitioners control over key environmental parameters that cannot be controlled at in situ nurseries like temperature, light, feeding, water quality, and algal competition. This control allows conditions that can maximize growth and survivorship of corals being reared for restoration^34–37^. Indeed, the growth benefits of ex situ rearing, including feeding corals to increase growth, have long been leveraged by the aquarium trade to increase coral production^36^. In our study, the 2020 cohort corals reared ex situ grew significantly faster than those grown in in situ nurseries, with colonies that received supplemental feeding growing at the fastest rate (Fig. 2a). For the 2021 cohort, the growth rate of Fed colonies was more than 2x greater than in the ex situ Unfed and In Situ treatments, highlighting the benefits of feeding corals during the 3 month ex situ rearing and pre-outplant phase (Fig. 2b). Heterotrophy can provide a substantial portion of coral nutrition, accounting for up to 2/3 of the carbon incorporated into coral skeletons and daily metabolic carbon demands^38,39^, as well as macro- and micronutrients that are not produced through photosynthesis. A recent review of how trophic interactions can be successfully incorporated into coral restoration highlighted the potential of optimizing coral feeding programs and the need for more research in this area^40^. Our study suggests that providing both a stable environment and supplemental feeding can help increase production of propagated corals.

Although likely more cost effective^36,41^, corals grown in situ can be impacted through numerous mechanisms including predation (this study), increased competition with algae^36^, and sub-optimal temperatures^37^. In turn, these impacts can reduce energy production or divert resources towards tissue repair and maintenance, ultimately reducing growth rates and coral survivorship^42–44^. For example, in our study nearly all of the 2020 cohort corals reared at the Broward nursery experienced predation that caused severe damage to these colonies, likely contributing to poor growth rates and the low (20%) survivorship of these colonies when outplanted to the reef (Fig. S1). These findings underscore the importance of site selection when planning the deployment of in situ coral nurseries.

For corals outplanted to reefs in Florida, predation by parrotfishes has emerged as a substantial bottleneck to survival, particularly for species with massive morphologies (i.e., non-Acroporid species)^17,18,28,29^. In the Caribbean, parrotfishes have been recorded targeting nutrient-rich prey, including reproductive polyps of corals^45^, as well as macroalgae^46 ^and seagrass^47 ^with elevated nitrogen content. Moreover, recent work in Florida suggests that corals with higher lipid and protein content experience increased predation when outplanted to reefs offshore of Miami, Florida USA^28^. Accordingly, we expected a tradeoff between the observed benefits of being conditioned under the ex situ Fed treatment (i.e. increased growth and protein content) with increased predation and related consequences like colony removal when corals were outplanted onto the reef. However, we observed the opposite; corals that performed best during the conditioning phase (ex situ corals) also had higher growth rates and survivorship after 3 months of being outplanted to the reef. These findings contrast those by Smith et al., who found that corals conditioned ex situ were more likely to be bitten by predators on reefs in the middle and upper Florida Keys than those conditioned in in situ nurseries. Similar to our findings, Toh et al. found that the growth benefits of feeding *P. damicornis* ex situ for five months before outplanting also translated to increased growth and survivorship for coral colonies outplanted to reefs in Singapore, though they did not report any predation on their outplanted corals. Beyond benefits to corals, they also found that feeding corals a high-density diet of *Artemia salina *nauplii was 12x more cost effective than not feeding corals on a per mm^3^of coral tissue basis^48^.

Although not tested as part of this study, pre-conditioning corals via supplemental feeding could potentially also benefit corals by increasing their ability to withstand additional stressors they are likely to encounter on the reef, such as thermal stress or disease. For example, modeling^49 ^and experimental work in the Caribbean^26,50 ^and Pacific^24^ has shown that corals with increased energy reserves, including protein and lipids, are better able to withstand and recover from thermal-stress induced bleaching. Similarly, pre-conditioning corals by exposing them to variable temperature regimes for short periods of time has been shown to increase the resistance of *Acropora cervicornis *to acute thermal stress^23^. However, recent acclimation experiments conducted over longer time scales (i.e., 6 years) have found that prolonged exposure to higher temperatures incurs a tradeoff in the form of decreased energy reserves^25^, highlighting the challenge of determining if and how to best acclimate corals for success when outplanted. Recent work has proposed that particular algal symbionts may confer some degree of influence to the susceptibility of SCTLD^51^ and is in part supported by field sampling that found that colonies of *Orbicella faveolata* affected by SCTLD tended to host the symbionts from the genus *Durisdinium *compared to colonies not affected by SCTLD that did not contain symbionts from this genus^52^. These findings suggest that pre-conditioning corals to host particular algal symbionts may be a possible method to increase the likelihood of coral recruit survival, but remains untested as a coral restoration strategy. Future research efforts investigating the potential benefits of pre-conditioning corals to increase their resistance to now common stressors like disease and thermal stress, including the duration for which these benefits last once outplanted to the reef, would benefit the development of optimal methods to propagate SCTLD-susceptible coral species for restoration.

We also observed that corals conditioned ex situ were less likely to be removed by predators than those conditioned in in situ treatments. Interestingly, although corals conditioned under the Fed treatment had higher protein content than in situ colonies after conditioning, there was no effect of conditioning treatment on the probability that a coral was bitten by a predator. Previous work in the Caribbean has also failed to find a relationship between the nutritional quality of coral tissue and predation by parrotfishes in some instances^53^. Indeed, the interplay between parrotfish and resource selection is mediated by numerous factors such as species identity and habitat type^54^, predator and territorial damselfish distribution, structural complexity^55^, and territoriality^56^, and cannot be solely attributed to resource quality. Following, one or a combination of these factors may have influenced our observed patterns in the incidence of predation (i.e. probability of being bitten; Fig. 5a) and overwhelmed any differences in resource quality from pre-outplant conditioning. Alternatively, it is possible that differences in resource quality among our treatments were not strong enough to elicit a detectable selective response by predators. However, we did find that corals conditioned in situ were more likely to suffer complete removal compared to corals conditioned ex situ (Fig. 5b). One plausible explanation for this pattern is that Fed and Unfed corals, which were held in ex situ conditions for three additional months, were able to devote more resources to better attach themselves to growing substrates during the conditioning phase. Indeed, both ex situ treatments grew significantly faster than In Situ corals, providing support for coral size as a basic physical mechanism contributing to our observed patterns in colony removal. Beyond stronger attachment because of increased surface area contacting the substrate, smaller corals also require less predation to experience complete removal compared to larger corals. For example, a single bite may be sufficient to consume and remove an entire small coral recruit, whereas numerous bites would be required to remove a larger coral. Alternatively, smaller recruits may be less easily detected by corallivores, and thus are less targeted compared to larger outplants, although our data do not suggest such an escape in size translated to increased survivorship for smaller (2021 cohort) corals. Other coral outplant studies in Florida have found that larger (25 cm^2^ live tissue) groupings of corals were less likely to experience complete mortality from predation compared to smaller (5 cm^2^live tissue) corals^28^. Coral size as a driver of outplanted coral removal or retention is further supported by our results documenting higher removal of the substantially smaller and younger 2021 cohort corals compared to the larger and older 2020 cohort corals (mean live area ± SE at time of outplanting: 0.41 cm^2^ ± 0.0003 vs. 5.66 cm^2^ ± 0.0009). Taken together, these findings highlight the importance of maximizing growth rates before outplanting and increasing the duration of ex situ rearing of sexual recruits to increase the likelihood of coral survivorship, and underscore predation as a key influence on the success of coral restoration efforts in locations like Florida, USA.

Predation on outplanted corals varied widely between regions, with the most intense predation observed at our site in Miami (Fig. 4). Variable levels of predation across such a large geographic range (> 200 km) are not particularly surprising, as substantial evidence demonstrates that corallivory varies on multiple spatial scales such as reef sites^57^, depths within a site^58^, and microhabitats within an individual coral bommie^59^. Although relative levels of predation incidence varied among sites, it is compelling that predation was most intense during the first week across all sites (Fig. 5), a finding consistent with recent studies focused on outplanting massive corals to Florida reefs^17,18^. Thus, the first several weeks after outplanting appear to be a critical bottleneck that smaller outplanted corals must survive to persist on Florida reefs. Indeed, Smith et al. found that although 53.8% of outplanted corals experienced some level of predation within the first week, nearly all corals (96%) recovered and were alive after 12 weeks. Thus, if outplanted corals can survive initial predation or detachment by predators, it appears that they may be able to recover and persist on these reefs. This is particularly relevant for places like Miami, where more than 85% of corals in our study were either bitten or removed within the first week of outplanting. Conversely, in places like Monroe and Broward, predation appears to be a lesser impediment to the restoration of massive corals. However, future studies that track longer term (i.e. > 3 months) impacts of initial predation on outplanted corals are necessary to fully understand the influence of corallivory on the success of coral restoration efforts. The wide variation in predation intensity among our sites underscores the importance of conducting field experiments that are replicated across regions to identify generalizable patterns and understand region-specific nuances. Following, investigating the relationship between predation on outplanted corals and likely drivers such as corallivorous fish communities and benthic community composition is needed to better inform coral restoration efforts.

As wild coral populations dwindle, introducing new sexual recruits may become increasingly important to maintain viable populations with the capacity to adapt to climate change^60^. Our findings have several key implications for the production and restoration of massive coral species using sexually propagated corals on reefs in Florida and throughout the Caribbean. When possible, rearing coral recruits at ex situ facilities with supplemental feeding can confer numerous benefits including increased growth rates and enhanced nutrition. Importantly, these pre-outplant benefits did not seem to incur any tradeoffs with predation when juvenile corals were outplanted to reefs, but rather translated to enhanced outplant success. This lack of a tradeoff appears to be mainly driven by coral size; larger corals are less likely to be removed by predation and thus are more likely to survive the removal bottleneck. However, ex situ coral rearing is not feasible in many locations, and presents numerous tradeoffs compared to rearing corals in situ, namely in scale (i.e. number of corals that can be propagated) and likely in investment (financial and time). Thus, efforts to integrate beneficial practices such as feeding into in situ propagation efforts could provide similar benefits to ex situ rearing and increase the success of coral outplanting efforts.

## Methods

### Study species

*Diploria labyrinthiformis *is a reef-building species found on reefs across the Western Tropical Atlantic with a mounding morphology that provides habitat for other marine organisms and contributes to the physical protection that shallow water ecosystems provide coastal communities^61,62^. *Diploria labyrinthiformis* is highly susceptible to SCTLD, with some populations experiencing disease in 30% of the colonies (Sharp et al., 2020). Once common on many reefs in Florida, *D. labyrinthiformis *colony density has now declined to < 25% of pre-SCTLD levels^3,8,63^. This study was conducted from November of 2021 through May of 2022 and consisted of two phases; (1) a pre-outplant coral conditioning phase, and (2) a post-conditioning coral outplant phase.

### Pre-outplant coral conditioning phase

Adult colonies of *D. labyrinthiformis* at The Florida Aquarium were housed in aquarium systems and conditioned for spawning as described for *M. meandrites* in O’Neil et al. Briefly, adult corals were housed in 1,250 L temperature controlled recirculating aquarium systems filled with pre-sterilized natural seawater and cultured live rock originating from Florida waters. Solar, lunar, and temperature profiles were programmed to mimic those experienced in Key Largo, FL. During the expected spawning windows, aquaria were monitored for the presence of gamete bundles being released, which contain both sperm and eggs and float to the surface. In May of 2020 and May of 2021, gamete bundles were collected from spawning individuals and a total of 11 parent colonies contributed to a mixed larval batch each year. Nine of the eleven parents were the same between each year, with two parent corals being different between the 2020 and 2021 cohorts. Larvae were settled onto conditioned ceramic tiles (Boston Aqua Farms, 3 cm × 3 cm; L × W) and reared in aquaria located in greenhouses at The Florida Aquarium Conservation Campus in Apollo Beach, Florida. Before the start of the experiment, recruits were housed in recirculating seawater aquaria in greenhouses at a water temperature of 25–28 °C. At approximately four months post-settlement, clustered recruits were manually separated from the ceramic tiles they had settled on using a razor blade and were attached to individual coral plugs (Boston Aqua Farms, 3 cm diameter) via superglue for further grow out. After this initial transfer, corals remained on these 3 cm diameter plugs for the duration of the pre-outplant conditioning phase and post-conditioning outplant phase described below to avoid introducing any confounding factors (e.g., injury) that may be incurred during the transfer of corals. Before initiating the experiment, and also in the Fed treatment, coral recruits were target fed 3–4 times weekly with a mix of Reef-Roids^®^ (Polyp Lab, USA), Oyster Feast^®^ (Reef Nutrition, USA), Real Oceanic Eggs™ (Reef Nutrition, USA; 2020 cohort only), and Golden Pearls Active Spheres (50–100 micron and 200–300 micron; 2021 cohort only). During feeding, water flow was turned off for one hour in the tray holding the corals and coral plugs were basted with a pipette filled with a mixture of the above items diluted into seawater.

On November 4 of 2021, 900 sexual recruits of *D. labyrinthiformis* were randomly selected from available 2020 (18 months post-settlement) and 2021 (6 months post-settlement) cohorts (*n* = 450 corals per cohort). Extreme outliers (i.e., particularly small or large corals) within each cohort were not considered for selection to standardize sizes within each cohort as much as possible. Upon selection, corals began conditioning under one of three treatments: (1) corals were kept in recirculating seawater systems at The Florida Aquarium’s ex situ facility and fed a diet of premade liquid and powdered invertebrate food as described above (henceforth “Fed”), (2) corals were kept at the Florida Aquarium’s ex situ facility under the same conditions as Fed corals but were not supplemented with any food (henceforth “Unfed”), or (3) corals were conditioned at in situ nurseries (henceforth “In Situ*”*) operated by Nova Southeastern University (NSU), the University of Miami (UM), or Mote Marine Lab (MML). Both in situ and ex situ nurseries can vary widely in numerous important parameters including flow, lighting, water quality (see nursery descriptions below) that may influence success on a species-specific basis. These treatments were selected to better understand the benefits and cons of rearing corals ex situ vs. in situ, as well as the potential value of investing additional resources in feeding corals during the rearing phase.

Nurseries were located offshore in Broward County (NSU), Miami-Dade County (UM), and Monroe County the Lower Florida Keys (MML), respectively (locations henceforth referred to by county names; Broward, Miami (i.e., Miami-Dade), and Monroe). Corals acclimated at the Broward nursery were located on mesh racks attached to coral propagated tables elevated ~ 1 m above the substrate, whereas corals acclimated at the Miami and Monroe nurseries were located on mid-water trees^64^. A total of 300 corals were conditioned under each treatment (*n* = 150 corals per cohort). For the In Situ treatment, 100 corals were conditioned in each nursery (*n* = 50 corals per cohort). Corals were conditioned under Fed and Unfed treatments from November 4 of 2021 until February 4 of 2022, for a total of 92 days of conditioning. Fed corals continued on the same diet described above for the pre-outplant conditioning phase. Corals were conditioned under In Situ treatments from November 4 of 2021 until February 7 at the Broward and Monroe nurseries, and until February 8 of 2022 at the Miami nursery, for a total of 95 and 96 days of conditioning, respectively.

Each coral colony was labeled to allow us to track individuals throughout the duration of the experiment. Calibrated, top-down photographs (Cannon PowerShot G1 × Mark II) of *D. labyrinthiformis *colonies were taken in water at the beginning (0 days) and end (92 days, 95 days, or 96 days) of the conditioning period. These images were processed in ImageJ^66^ to quantify the planar area (cm^2^) of live tissue for each individual coral colony.

## Post-conditioning coral outplant phase

### Post-conditioning coral outplant phase

At the conclusion of the conditioning period, colonies of *D. labyrinthiformis* conditioned under the ex situ treatments at The Florida Aquarium were transported to land-based nurseries at NSU, UM, and MML in coolers filled with fresh seawater. At each location, corals were kept in flow-through seawater systems until outplanting occurred on February 7 (Monroe) and February 8 (Broward and Miami) of 2022. At the time of outplanting, corals from the 2020 cohort were 21 months of age and corals from the 2021 cohort were 9 months of age.

Corals were outplanted to a single reef site within each region (Fig. 1). All sites were in approximately 3–6 m depth of water and were chosen based on site familiarity and the success of previous coral restoration efforts focused on massive coral species^13,17^. Within a site, corals were outplanted into plots approximately 50 × 30 cm in size, with each plot separated from each other by at least 1 m. Plots at sites in Miami and Broward were arranged along multiple linear transects as needed to keep all plots within the same reef type. Plots in Monroe were located on top of dead massive coral colonies interspersed among a single high-rugosity patch reef site. Each plot received six coral outplants and contained one coral from each of the cohort (2020 or 2021) and conditioning (Fed, Unfed, or In Situ) treatment combinations. Corals within a plot were placed 15 cm apart in a 3 × 2 grid and were secured individually to the reef substrate using cement (Broward and Miami) or epoxy (Monroe). Corals were deployed in one of nine predetermined orientations to randomize the position of the different cohort × treatment corals within plots. A total of 752 colonies of *D. labyrnthiformis* were outplanted in February of 2022 across our three sites. The number of corals from each cohort outplanted to each region varied based on survivorship of corals during the coral conditioning period (Table S1). The mean live area ± SE at time of outplanting was 0.41 cm^2^ ± 0.0003 for the 2021 cohort corals and 5.66 cm^2^ ± 0.0009 for the 2020 cohort corals (Fig. S3).

We surveyed outplanted colonies after 1 day, 1 week, 2 weeks, 1 month, 2 months, and 3 months of deployment. During each survey, we recorded if a colony was alive or dead (i.e., survivorship). Corallivorous fishes leave distinct bite marks on coral colonies that are easily distinguishable from other sources of mortality^44,65^. Therefore, we also recorded if a colony was unbitten, displayed signs of fish predation, or completely removed. Removal by predators was attributed to corals where there were obvious visual cues, i.e., bite marks on the plug and surrounding area where the coral was previously located, indicating that predation was the cause of coral removal (Fig. S4). There were no instances of plugs being detached or removed from the substrate during the course of the experiment. Corals had been growing on these plugs for 5–19 months before they were deployed to the field, allowing sufficient time to attach themselves to the ceramic plugs via growth and thus making it extremely unlikely that removal would occur via water flow or other abiotic factors experienced at outplant sites. In addition to in situ surveys, we took a calibrated photograph of each plot and colony on the day of outplanting (0 days) and again at the end of the experiment approximately 3 months after deployment (Broward = 92 days; Miami = 96 days; Monroe = 95 days). All images were processed in ImageJ^66^ as described previously.

## Lipid and protein analyses

To understand if- and how coral tissue metrics correlate with coral survival and predation intensity, *D. labyrinthiformis* lipid and protein content was quantified before (*n* = 25 samples from each cohort) and after conditioning (*n* = 10 samples per cohort per treatment). Frozen samples were packed on dry ice and shipped overnight to Florida State University’s Coastal and Marine Lab (CML). At the CML, the corals were inventoried, photographed, and immediately stored at − 80 C to await preparation and analysis.

Before processing, all epiphytes, fouling organisms were removed by scrubbing the plugs with a wire brush. After cleaning visible portions of the coral plugs, excess epoxy was cut away from the colonies with a diamond-coated bandsaw (Gryphon Aquasaw, Gryphon Corporation, Sylmar CA, USA). The resulting frozen coral colonies were then placed in a Retsch Mixer Mill 400 (Verder Scientific, Newton PA, USA) and ground for 60 s each in liquid nitrogen cooled mixing jars to obtain a homogenous paste tissue slurry. Lipid content was determined gravimetrically using the Folch method^67 ^modified for corals by Mclachlan et al^68^. Briefly, ~ 1000 mg sample^−1^ of the frozen ground tissue slurry, weighed to the nearest milligram, was transferred to sample vials, lyophilized for 12 h at − 50 C, then sealed and stored sealed at − 80 C until extraction. For extraction, freeze-dried samples were mixed with a 2:1 chloroform: methanol solution containing butylated hydroxytoluene to prevent oxidation, and incubated in the dark for 60 min. After incubation, the samples were filtered through pre-weighed and combusted gf/f filters. The filters were dried to a constant weight at 55 C, combusted at 450 C, and reweighed to determine each sample’s organic content while the filtrate was loaded into separatory funnels, rinsed with 4 ml of 0.88% KCl, and allowed to separate in the dark. After separation, the lipid portion of the filtrate was drained into pre-weighed and combusted flasks, mixed with ~ 1 ml of methanol, evaporated under a steady stream of nitrogen, and reweighed to determine the lipid mass.

Protein content was analyzed from the same tissue slurry using a modified Bradford assay^69^ For each sample, approximately 500 mg of tissue was weighed and mixed with a 1× solution of Radioimmunoprecipitation (RIPA). Cells were lysed through three repeated freeze-thaw cycles by dipping the sample in liquid nitrogen followed by submersion in a room temperature water bath. After the third cycle, samples were centrifuged for 20 min at 4200 rpm (4122×*g*). The supernatant from each sample was then collected and analyzed at 595 nm on BioTek Synergy Multimode microplate reader (Agilent, Santa Clara CA. USA) using Bio-Rad protein assay kits following the manufacturers protocol.

Samples from the 2021 cohort were too small to provide sufficient tissue to analyze both lipids and protein. Therefore, corals from this cohort reared in the same treatments and rearing locations were pooled until at least 1000 mg of tissue was available for lipid analysis. Pooling tissue provided enough tissue for lipid analysis for two replicates from the Unfed, Broward, and Monroe In Situ treatments and 3 replicates from the Fed, and Miami In Situ treatments.

### Statistical analyses

Individual daily absolute growth rates (cm^2^ day^−1^) were calculated by subtracting the initial size (live area; cm^2^) from the final size and dividing by the number of days elapsed. This was done for the pre-outplant conditioning phase and the post-conditioning outplant phase. Only corals that were alive at the final time point were included in growth rate analyses. We also calculated growth rates normalized to the initial area of live tissue by dividing the absolute growth rate by the initial colony size to provide a daily growth rate normalized to initial coral size. Normalized growth rates were calculated as these account for the initial size of a coral and allow an assessment of how much new live coral tissue was produced under each treatment based on the starting amount of live coral tissue. We assessed differences in absolute and normalized growth rates during the pre-outplant conditioning phase using a linear mixed effects model that considered Cohort and Treatment as fixed interacting factors and Location as a random effect (intercept). We excluded corals from the Broward In Situ nursery from growth rate analyses because they suffered high rates of predation during the conditioning phase. For both absolute and normalized growth rate models, we used a weighted variance structure to allow variance to differ between cohorts.

We tested differences in final lipid (% dry weight) and protein (mg g^−1^) content using one-way Analysis of Variance tests that contained five levels: (1) Ex Situ Fed, (2) Ex Situ Unfed, (3) Monroe In Situ, (4) Miami In Situ, and (5) Broward In Situ. For these analyses, we tested for differences in protein or lipid content within individual cohorts (i.e. 2020 or 2021). There was insufficient tissue to analyze final protein content for the 2021 cohort corals.

We assessed for differences in the probability of corals being bitten or removed by predators using generalized linear models (glm) with a binomial distribution. These models considered Survey, Location, Cohort, and Treatment as fixed factors and included a Cohort × Treatment interaction, with Wald Chi Square tests to assess global significance. Individual models were used to assess the probability of a coral being (1) bitten or (2) removed during the course of our field experiment. We used a glm with the same structure and factors to test the effects of outplant Location, Cohort, and Treatment on the probability of a coral being alive (i.e., survivorship) at the end of the experiment. We assessed differences in absolute and normalized growth rates during the post-conditioning outplant phase using two-way ANOVAs that considered Treatment and Location as fixed interacting factors. For these analyses, we analyzed each cohort individually because of large differences in residual variance.

All analyses were conducted in R version 4.2.2^70 ^with general data manipulations and plotting conducted using the R package tidyverse^71^. Generalized linear and mixed effects models were conducted using the R package glmmTMB^72 ^and residuals and assumptions were visualized and checked using DHARMa^73^. When significant main effects or interactions were found for any of the models, we conducted post hoc tests with Tukey’s correction using the R package emmeans^74^.

## Electronic supplementary material

Below is the link to the electronic supplementary material.

Supplementary Material 1

## Data Availability

Data availability statement Raw data will be freely available upon request. Data requests should be directed to the corresponding author via the provided email address.
