# Supplementary material for: Acclimation and size influence predation, growth, and survival of sexually produced Diploria labyrinthiformis used in restoration
Source: Sci Rep. 2024 Nov 1;14:26362. doi: 10.1038/s41598-024-73727-8 (PMC11530667; doi:10.1038/s41598-024-73727-8)
Supplement: Supplementary file 1 — Supplementary Material 1 [file 41598_2024_73727_MOESM1_ESM.docx]

**Table S1**: Total number of colonies of each cohort outplanted to the three study sites.

|  | **Cohort** | |  |
| --- | --- | --- | --- |
| **Location** | **2020** | **2021** | **Total** |
| Broward | 129 | 116 | 245 |
| Miami | 133 | 124 | 257 |
| Monroe | 133 | 117 | 250 |


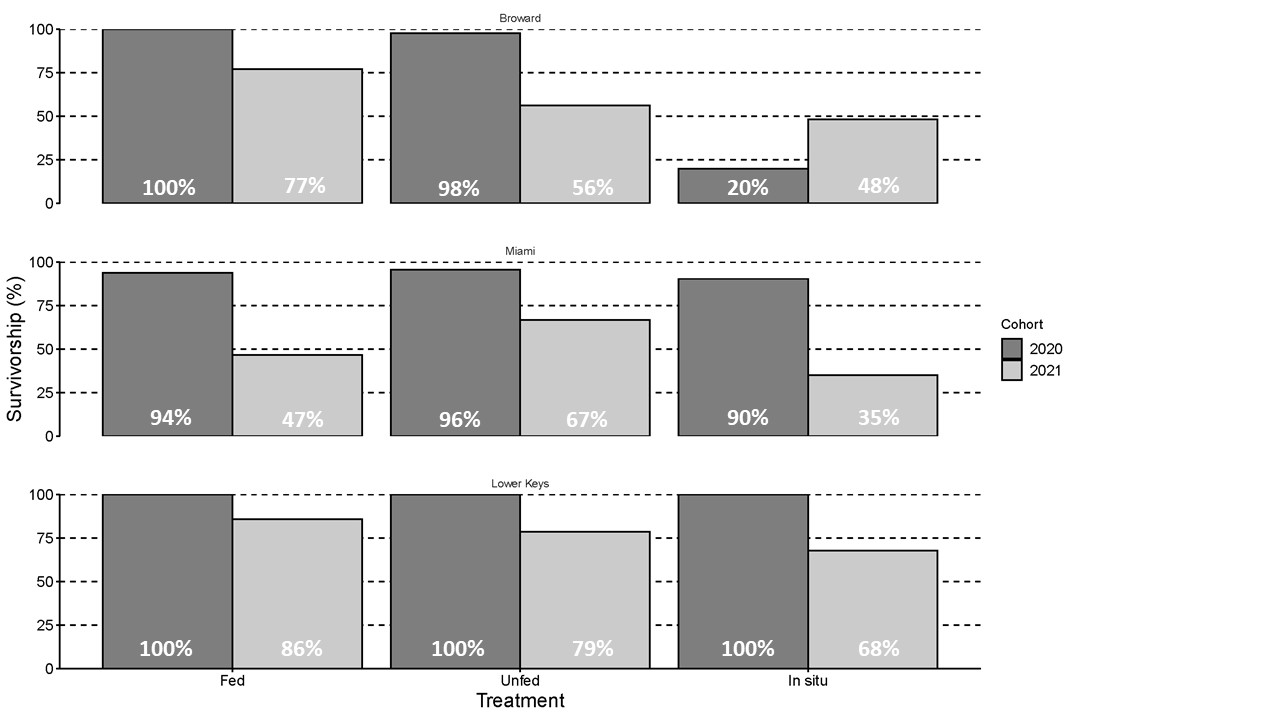


**Figure S1**. Survivorship for each Cohort x Treatment combination within each Location at the end of the three-month outplant period.

**Figure S2.** Mean absolute growth rate (cm^2^ day^-1^) of *D. labyrinthiformis* colonies after three months of being outplanted to reef sites in South Florida. Statistics from generalized linear mixed effects model. Bars that share the same letter indicate the lack of a statistically significant differences between values within a location per *post hoc* test with Tukey’s correction.

**Figure S3**. Boxplot of initial coral sizes (cm^2^ live tissue) used in the outplant phase of the experiment. Bold horizontal lines represent median values, upper and lower hinges correspond to 1^st^ and 3^rd^ quartiles.


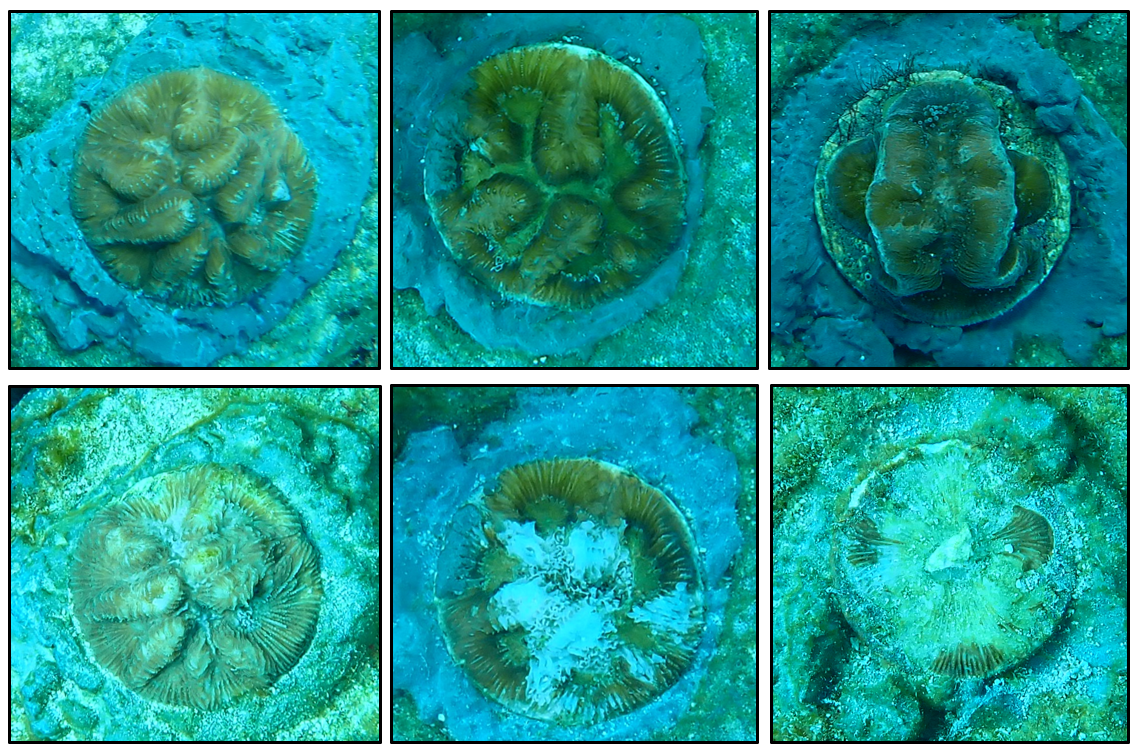


**Figure S4**. Examples of 2020 cohort *D. labyrinthiformis* colonies display signs of no predation (left column), moderate predation (middle column), and near-complete predation (right column).
